# Popliteal artery aneurysms treatments: early midterm results of the use of endovascular stent grafts

**DOI:** 10.3906/sag-2005-263

**Published:** 2021-06-28

**Authors:** Kaptanıderya TAYFUR, Mehmet Şenel BADEMCİ

**Affiliations:** 1 Department of Cardiovascular Surgery, Faculty of Medicine, Ordu University, Ordu Training and Research Hospital, Ordu Turkey; 2 Department of Cardiovascular Surgery, Faculty of Medicine, İstanbul Medeniyet University, Göztepe Training and Research Hospital, İstanbul Turkey

**Keywords:** Peripheral, vascular, stenting, aneurysm

## Abstract

**Background/aim:**

Popliteal artery aneurysms (PAAs) are abnormal bulgings, which account for 70% of all peripheral artery aneurysms. They are usually asymptomatic. In this study, we present our long-term results of endovascular stent grafts in the treatment of PAA in the light of literature data.

**Material and methods:**

A total of 63 legs of 63 patients with PAA, who were treated with endovascular techniques in our clinic between July 2010 and July 2019, were retrospectively analyzed. All patients underwent color Doppler ultrasound (DUS), magnetic resonance angiography (MRA), or computed tomography angiography (CTA) to identify the diameter and length of PAAs, vessel tortuosity, the presence and degree of thrombus, and diameter in the healthy landing zone and to visualize tibioperoneal vascular structures. A Viabahn stent graft was inserted in all patients.

**Results:**

57 patients (90.5%) were males with a mean age of 76.35 **±** 7 years. 24 patients (38.1%) were symptomatic, while 11 patients (17.5%) had a concomitant abdominal aortic aneurysm (AAA). The mean follow-up period was 46.05 **±** 25.01 months. The primary patency rate was 79.3%. A graft thrombosis was observed in 13 patients (20.6%) during a mean follow-up period of 8.31 **±** 5.91 months. The number of distal arteries was significantly lower in the patients with thrombosis than those without.

**Conclusions:**

Endovascular treatment of PAA using stentgrafts is safe in selected cases. However, it is reasonable to avoid endovascular treatment due to an increased risk for thrombosis in patients with a low number of patent distal arteries or impaired distal flow.

## 1. Introduction

Popliteal artery aneurysms (PAAs) are abnormal bulgings, which occur in the wall of the blood vessel and account for 70% of all peripheral artery aneurysms. The prevalence of PAAs has been estimated as 1% in men aged between 60 and 80 years. [1] They mostly affect male sex with a male-to-female ratio of 20:1. [2] The majority of PAAs are asymptomatic and diagnosis is usually made based on the presence of a wide arterial pulse in the popliteal fossa region on palpation during physical examination or coincidentally by imaging modalities used for other reasons.

The natural course of the disease includes both acute and chronic thromboembolic complications. Nearly half of patients with chronic thromboembolism present with intermittent claudication, rest pain, and/or acral necrosis. [3] Although PAA rupture is uncommon, acute thrombosis and distal embolization may be present in 30% of cases, increasing the amputation rate up to 20% [4,5]. Irrespective of the aneurysm diameter, all symptomatic PAAs and asymptomatic cases with an aneurysm diameter of >2 cm along with a severe mural thrombus and poor infrapopliteal arterial run-off are recommended to be treated. [6]

Doppler ultrasound (DUS), magnetic resonance angiography (MRA), computed tomography angiography (CTA), and conventional angiography are the most commonly used diagnostic tools. Surgical repair with a saphenous vein graft or synthetic graft is the gold standard in the treatment of PAAs. [6] However, after the first successful endovascular repair of a ruptured abdominal aortic aneurysm (AAA) was performed by Marin et al. [7] in 1994, endovascular treatment has been increasingly adopted among surgeons, particularly for high-risk patients for surgery and for those refusing a surgical intervention. The main advantages of endovascular repair include shorter operation duration, potential avoidance of general anesthesia, possibility of bilateral intervention in a single session, shorter length of hospital stay, and less perioperative mortality [8,9].

On the other hand, no consensus on endovascular treatment of PAAs has been reached, yet. In this study, we present our long-term results of endovascular stent grafts in the treatment of PAA and to provide in the light of literature data.

## 2. Materials and methods

### 2.1. Study design and study population

This single-center, retrospective study included a total of 63 legs of 63 patients with PAA who were treated with endovascular techniques in our clinic between July 2010 and July 2019. Data including demographic and clinical characteristics and aneurysm and operative data were recorded. Asymptomatic patients with an aneurysm diameter of >20 mm were also included. The main complaints of symptomatic patients on admission include persistent knee pain, venous pressure symptoms, claudication, and bulging behind the knee joint. A written informed consent was obtained from each patient. The study protocol was approved by the local Ethics Committee. The study was conducted in accordance with the principles of the Declaration of Helsinki.

**Figure 1:**
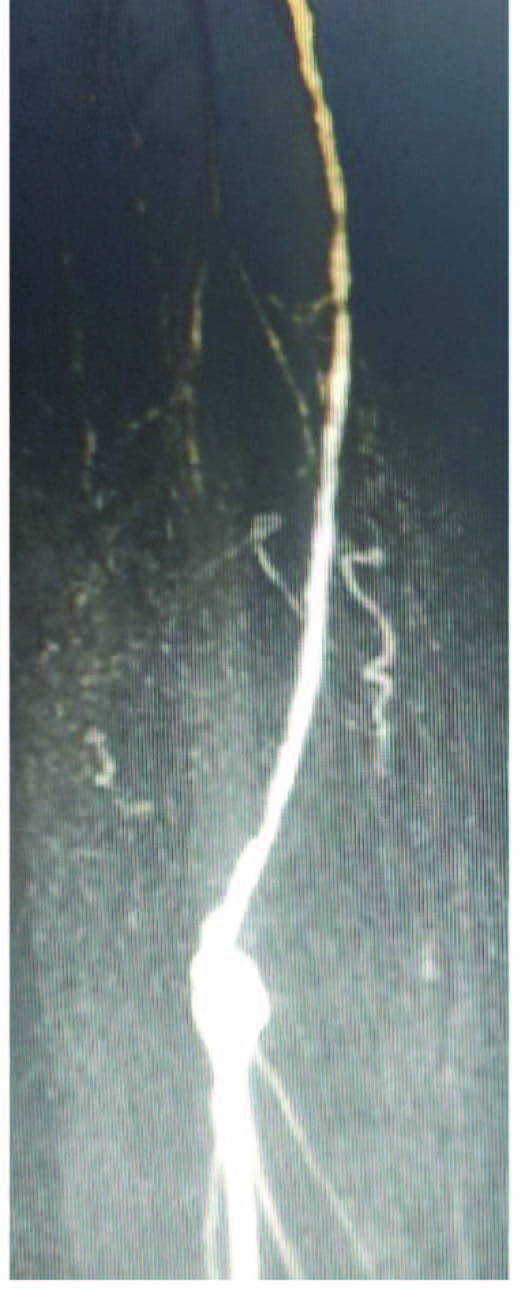
Preoperative magnetic resonance angiography image showing a popliteal aneurysm.

All patients underwent color DUS, MRA, or CTA to identify the diameter and length of PAAs, vessel tortuosity, the presence and degree of thrombus, and vessel diameter in the healthy landing zone and to visualize tibioperoneal vascular structures (Figure 1). Patients with severe atherosclerotic occlusive disease at the level of iliac and femoral arteries, without an adequate and appropriate distal and proximal landing zone, and those with unsuitable tibioperoneal anatomy were considered ineligible for endovascular treatment and excluded.

### 2.2. Endovascular technique

**Figure 2:**
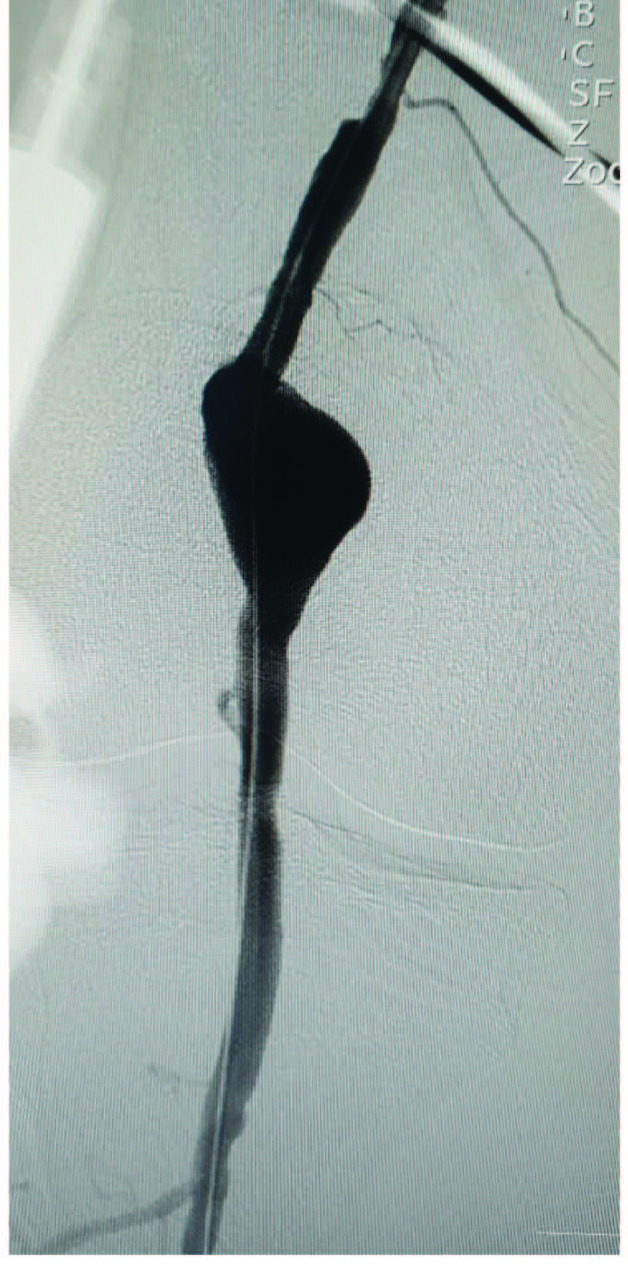
A preoperative conventional angiography image showing a popliteal aneurysm.

**Figure 3:**
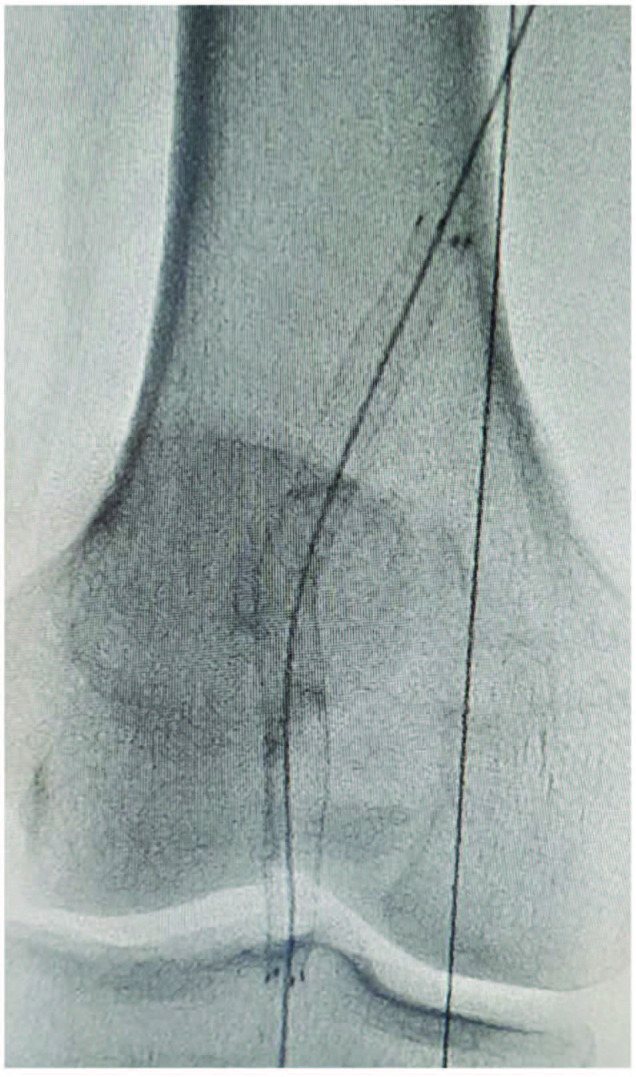
A Viabahn stent graft was inserted using a 0.035-inch guidewire.

**Figure 4:**
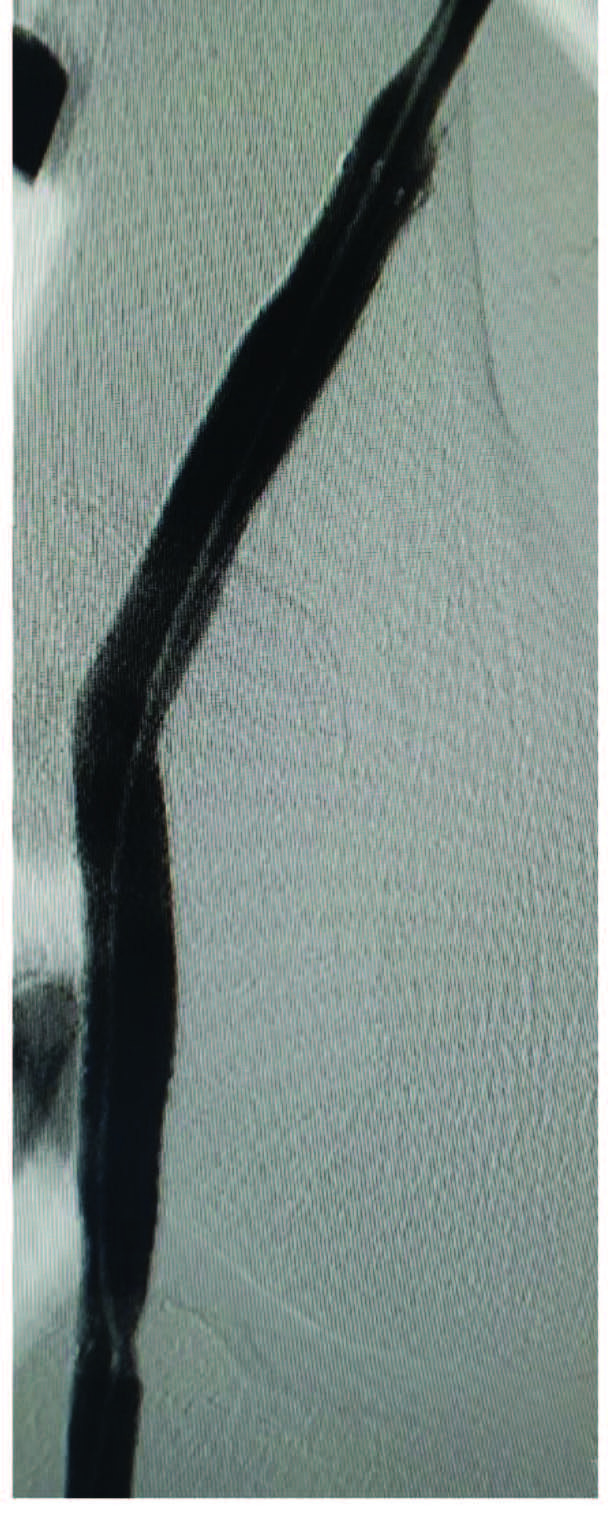
Postoperative elimination of aneurysm.

All procedures were performed under spinal or local anesthesia in the angiography laboratory by experienced surgeons. A 7Fr or 9Fr sheath was inserted to the femoral artery through percutaneous access, while ≥10Fr sheath was reserved for surgical access. All patients received 5000 IU standard heparin after the procedure. Computed tomography angiography was performed to identify the size and location of PAA and vessel size and diameter in the healthy landing zone (Figure 2). A 0.035-inch guidewire was then inserted into the vessel lumen for stent graft implantation. A Viabahn stent graft (W.L. Gore & Associates Inc., Flagstaff, AZ, USA) was inserted in all patients (Figure 3). The diameter and length of the stent graft to be used were chosen based on precise MRA or CTA measurements preoperatively. The diameter of the graft to be used was selected as exceeding the luminal diameter by 10 to 15%. For the use of multiple stent grafts, distal stents were placed initially and proximal stents were then placed, providing an overlap by a minimum of 20 mm. After stenting, balloon dilatation was performed. Technical success was defined as the elimination of aneurysm without endoleak and with intact distal flow (Figure 4). Following the procedure, a loading dose of clopidogrel 300 mg was given to all patients, followed by 75 mg clopidogrel and 100 mg acetylsalicylic acid daily. Treatment modification was avoided in patients using oral anticoagulants for other indications. For this technique, appropriate proximal and distal stent landing zones and patent distal tibioperoneal arteries should be met. And also, increased aneurysm length is a disadvantage for endovascular repair. The diameter and length of the stents should be chosen carefully. During the postoperative follow-up period, it should be ensured that patients receive correct and sufficient antiaggregant treatment.

### 2.3. Postoperative follow-up

All patients were followed at one, six, and 12 months for the first year and annually thereafter using CTA. During follow-up, stent graft fractures and graft thrombosis were evaluated through conventional angiography for patients with symptoms of thrombosis or claudication, and secondary interventions were planned. All patients were given 75mg clopidogrel and 100mg acetylsalicylic acid during the postoperative period.

### 2.4. Statistical analysis

Statistical analysis was performed using SPSS for Windows version 17.0 software (SPSS Inc., Chicago, IL, USA). Descriptive data were expressed in mean ± standard deviation (SD), median (interquartile range), or number and frequency. The Fisher’s exact test and Chi-square test were used to analyze significant differences between variables. The Kolmogorov–Smirnov test was used to test whether the variables were normally distributed. An independent samples t-test was used to determine whether there was a statistically significant difference in normally distributed data, while the Mann–Whitney U test was carried out for nonnormally distributed data. A P value of <0.05 was considered statistically significant with 95% confidence interval (CI). 

## 2. Results

Of all patients, 57 (90.5%) were males and six (9.5%) were females with a mean age of 76.35 **±** 7.46 (range, 45 to 88) years. At the time of admission, 24 patients (38.1%) were symptomatic, while 11 patients (17.5%) had a concomitant AAA (abdominal aortic aneurysm). Baseline demographic and clinical characteristics of the patients are shown in Table 1.

**Table 1:** Baseline demographic and clinical characteristics of the patients.

Variable	n (%)
Male	57 (90.5)
Age, year (mean ± SD)	76.35 ± 7.46
Concomitant AAA	11 (17.5)
Hypertension	44 (69.8)
Smoking	51 (81.0)
Diabetes	9 (14.3)
CAD	18 (28.6)
Hyperlipidemia	39 (61.9)
COPD	16 (25.4)
Symptomatic	24 (38.1)
PAA diameter, mm (mean ± SD)	32.19 ± 5.98
Aneurysm length, mm (mean ± SD)	228.97 ± 40.24
Number of distal arteries/patient (mean ± SD)	2.35 ± 0.72

The mean PAA diameter was 32.19 **±** 5.98 mm. Endovascular procedure was performed under local anesthesia in 53 patients, and access was maintained through percutaneous route in 50 patients. The mean number of stent grafts implanted was 1.55 **±** 0.64. None of the patients had any access site-related complications. Intraoperative data are shown in Table 2.

**Table 2:** Intraoperative data.

Variable	N (%)
Percutaneous access	50 (79.36)
Ipsilateral access	58 (92.06)
Local anesthesia	53 (84.12)
Contrast level (mean ± SD)	47.30 ± 10.66
Number of stents/patient (mean ± SD)	1.55 ± 0.64
Length of stent, cm (mean ± SD)	9.05 ± 3.45
Operation duration , min( mean ± SD)	39.51 ± 7.44
Technical success	63 (100)

The mean length of hospital stay was 1.92 ± 0.72 days. No in-hospital mortality was seen, and no stent graft thrombosis, migration, or fracture were observed within 30 days. The mean follow-up was 46.05 ± 25.01 months. The primary patency rate was 79.3%. A graft thrombosis was observed in 13 patients (20.6%) during a mean follow-up of 8.31 ± 5.91 months. Two patients (15.3%) with graft thrombosis had stent graft fracture, while five patients (7.9%) had thrombosis due to antiplatelet therapy cessation. The patients with stent graft fracture underwent a second intervention using additional stent grafts. However, five patients with graft thrombosis in whom fibrinolysis therapy failed underwent femoropopliteal bypass. Complete patency was achieved in patients undergoing additional stent graft insertion and fibrinolysis therapy. None of the patients needed amputation; however, five patients died from several reasons and were excluded from the follow-up. The secondary patency rate was 84.12%. No endoleak was observed during the follow-up (Table 3). 

**Table 3:** Postoperative data.

Variable	n (%)
Follow-up, month (mean ± SD)	46.05 ± 25.01
Primary patency	50(79.3)
Graft thrombosis	13 (20.6)
Time to thrombosis, month (mean ± SD)	8.31 ± 5.91
Bypass	4(6.3)
Stent graft fracture	2(3.2)
Secondary patency	53(84.12)
Mortality	5(7.93)
Limb loss	0
Length of hospital stay, day (mean ± SD)	1.92 ± 0.72

The mean PAA diameter was significantly higher, and the mean number of tibioperoneal distal arteries was significantly lower in the patients with graft thrombosis than those without (P < 0.05). The mean number of distal arteries was 1.77 ± 0.83 and 2.50 ± 0.61 in the patients with and without graft thrombosis, respectively (P < 0.05). However, there was no significant difference in other variables between the patients with and without graft thrombosis (Table 4).

**Table 4:** Comparison of demographic and clinical characteristics between patients with and without graft thrombosis.

Variable	Without thrombosis n = 50	With thrombosis n = 13	P
Male	45 (90.9)	12 (92.3)	0.639a
Age, year (mean ± SD)	77.14 ± 7.87	73.71 ± 4.66	0.099b
Abdominal aneurysm	8 (16.0)	3 (23.1)	0.405a
HT	34 (68.0)	10 (76.9)	0.398a
Smoking	40 (80.0)	11 (84.6)	0.528a
DM	7 (14.0)	2 (15.4)	0.599a
CAD	14 (28.0)	4 (30.8)	0.547a
Hyperlipidemia	30 (60.0)	9 (69.2)	0.392a
COPD	12 (24.0)	4 (30.8)	0.430a
Contrast level, cc (mean ± SD)	48.00 ± 9.90	44.61±13.30	0.312b
Aneurysm diameter, mm (mean ± SD)	20.10 ± 3.74	33.00 ± 6.21	0.007b
Aneurysm length, mm (mean ± SD)	229.65 ± 41.01	226.37 ± 38.59	0.796b
Number of distal arteries/patient [median (interquartile range)]	3(1)	2(1.50)	0.003c
Number of stents [median (interquartile range)]	1(1)	2(1)	0.122c
Length of stent, mm [median (interquartile range)]	9(5)	10(4)	0.867c
Follow-up duration, month [median (interquartile range)]	36(31.50)	70(57)	0.008c
Operation duration, min (mean ± SD)	40.70 ± 7.07	34.92 ± 7.31	0.011b
Length of hospital stay, day [median (interquartile range)]	2(0.25)	2(1)	0.412c

The mean PAA diameter and length and the mean number of stent grafts per patient were significantly higher in the patients with AAA than those without (P < 0.05). The mean PAA diameter was 37.98 ± 7.33 mm and 30.97 ± 4.91 mm in the patients with AAA and those without AAA, respectively (P < 0.05). In addition, the ratio of symptomatic patients with AAA was significantly higher than those without AAA (P < 0.05). All patients were intravenously hydrated using 0.9% isotonic saline after the procedure. None of the patients had acute renal insufficiency (Table 5).

**Table 5:** Comparison of demographic and clinical characteristics between patients with and without abdominal aortic aneurysms.

Parameter	Without AAA n = 52	With AAA n = 11	P
Male	49 (94.2)	8 (72.7)	0.060a
Age, year (mean ± SD)	75.88 ± 7.62	78.54 ± 6.52	0.286b
Thrombosis	10 (19.2)	3 (27.3)	0.405a
HT	34 (65.4)	10 (90.9)	0.089a
Smoking	42 (80.8)	9 (81.8)	0.652a
DM	7 (13.5)	2 (18.2)	0.495a
CAD	12 (23.1)	6 (54.5)	0.046a
Hyperlipidemia	28 (53.8)	11 (100.0)	0.003a
COPD	11 (21.2)	5 (45.5)	0.100a
Symptomatic	14 (26.9)	10 (90.9)	0.000a
Contrast level, cc (mean ± SD)	46.83 ± 11.29	49.54 ± 6.87	0.447b
Aneurysm diameter, mm (mean ± SD)	30.97 ± 4.91	37.98 ± 7.33	0.000b
Aneurysm length, mm (mean ± SD)	222.05 ± 35.50	261.66 ± 46.75	0.002b
Number of distal arteries/patient [median (interquartile range)]	3(1)	2(1)	0.222c
Number of stents [median (interquartile range)]	1(1)	2(2)	0.046c
Length of stent, mm [median (interquartile range)]	8(3)	15(10)	0.134c
Follow-up duration, month [median (interquartile range)]	36(34.50)	66(50)	0.059c
Operation duration, min (mean ± SD)	39.31 ± 7.14	40.45 ± 9.07	0.646b
Thrombosis duration, month (mean ± SD)	8.30 ± 6.13	8.33 ± 6.35	0.994b
Length of hospital stay, day [median (interquartile range)]	2(0)	2(1)	0.143c

When the 63 patients included in the study were analysed, 6mm stentgraft was used in 30 patients, 7mm stentgraft was used in 22 patients, and 8mm stentgraft was used in 11 patients. In all patients, 1 mm larger balloon than stent graft was used for fixation and dilation. In the study, stent thrombosis was seen in 13 patients. In 9 of 13 patients,had 6mm stentgraft and in the remaining 5 patients had 7mm stent graft with thrombosis.

## 3. Discussion

Popliteal artery aneurysms are the most common peripheral artery aneurysms with an estimated incidence of 0.1 to 2.8% [10]. About 80% of PAAs asymptomatic at the time of diagnosis and are often diagnosed incidentally; however, asymptomatic PAAs become symptomatic at a rate of 14% per year. [11] Symptomatic PAA may resemble acute or chronic limb ischemia, knee pain, nerve entrapment or vein pressure. In general, an aneurysm diameter of >20 mm is an indication for surgery in asymptomatic cases [12]. Ruptured or symptomatic PAAs should be treated immediately. About 20% of asymptomatic cases are at risk for limb loss due to late complications of PAA [13].

In the literature, there is still no consensus on the optimal treatment of PAA. Conventional treatment includes bypass surgery using an autologous saphenous vein or synthetic graft. However, access-related complications may occur in 30 to 40% of cases, indicating a high perioperative morbidity rate [14]. In addition, surgery is associated with postoperative complications such as major amputation, major bleeding requiring a second operation, nerve injury, deep vein thrombosis or wound site infection [15–16]. Therefore, endovascular technique offers more advantages than conventional surgery in the treatment of PAA with shorter operation duration and length of hospital stay, more rapid functional recovery, minimal blood loss and wound site infection, and avoidance of general anesthesia. Endovascular treatment, however, is associated with stent graft occlusion, fracture, or migration and endoleak; thus, it is still controversial in the treatment of PAA. Popliteal artery aneurysms may be bilateral and most of them present with aneurysms of other anatomic locations. They are mostly seen in men [6]. In our study, 90% of the patients were males and 17.5% of the patients had a concomitant AAA. Of note, Dick Cheney, the vice president of the United States, underwent endovascular stent grafting for bilateral PAA in 2005 [17]. In the same year, Antonella et al. published the first prospective, randomized study comparing open surgery versus endovascular technique in the treatment of PAA [14]. The authors found no statistically significant difference in the four-year patency rates and limb salvage between the two groups. Nonetheless, the authors emphasized the advantages of endovascular treatment with shorter length of hospital stay and rapid recovery. In another study, the primary and secondary patency rates were found to be 84.8% and 96.8%, respectively without limb loss [18]. Piazza et al. [19] evaluated 47 patients with PAA and reported a primary and secondary patency rate of 76% and 82%, respectively using endovascular technique. Consistent with the literature, the primary and secondary patency rates were 79.3% and 84.12%, respectively in our study.

The popliteal region is constantly subjected to movement and bending which may endanger of the performance and durability of the stent graft. Therefore, the stent graft to be chosen is of utmost importance. It should be very flexible and resistant. In a study, heparin-coated stent grafts yielded a significantly higher patency rate than bare metal stents in the treatment of PAA [20]. Similarly, the Viabahn stent graft is a composite based on the combination of a thin, heparin-coated polytetrafluoroethylene lining and a surrounding self-expanding nitinol stent. In our study, a Viabahn stent graft was inserted in all patients. Furthermore, a successful stent graft implantation requires appropriate proximal and distal landing zones and patent distal tibioperoneal arteries [21]. The evaluation of the distal arteries is critical to ensure the durability of the endovascular repair, since the number of patent distal arteries increases, the possibility of stent occlusion decreases. The incidence of graft thrombosis has been reported to be higher in patients with only one patent distal artery than those with two or more patent arteries [22]. In our study, the limited number of distal arteries was significantly correlated with an increased rate of graft thrombosis. The mean number of distal arteries was 1.77 ± 0.83 and 2.50 ± 0.61 in the patients with and without graft thrombosis, respectively. In a recent study, the limited number of patent distal arteries was found to be an independent risk factor for poor primary patency rates. [23]

With the introduction of reports of Tielliu et al. [24] in 2005, the use of postprocedural antiplatelet therapy has become standard and has dramatically increased the patency rates. In our study, five patients who discontinued antiplatelet therapy within the first year developed graft thrombosis. In addition, PAAs of <30 mm have a lower complication rates [25]. Similarly, the mean PAA diameter was significantly higher in the patients with graft thrombosis in our study. It has been also well established that increased aneurysm length is a disadvantage for endovascular repair as it requires the use of more stent grafts. In our study, although we used a higher number of stent grafts in the patients with graft thrombosis, no significant difference was found. 

Review of the literature reveals limited data regarding the relationship between PAAs and AAAs. In our study, 11 patients had a concomitant AAA. The mean PAA diameter and length were significantly higher and the number of stent grafts used was significantly greater in the patients with concomitant AAAs than those without. In addition, 10 of 11 patients (90.9%) were symptomatic, which reached statistical significance.

Endovascular treatment of PAAs is associated with serious complications, such as endoleak caused by reflux of the blood into the aneurysmal sac through genicular arteries. The incidence of such endoleaks is very low in the literature [26–27]. Similarly, none of our patients had endoleak. In addition, similar to our study, the rate of stent occlusion has been estimated as low varying between 9 to 20% [18,22,28]. Graft thrombosis can be managed by a second endovascular intervention, fibrinolytic therapy, or surgical bypass. In general, stent graft thrombosis is associated with a high rate of amputation; however, none of our patients had limb loss, although the rate of graft thrombosis was 20.6%, treated with a second endovascular procedure. In a previous study comparing open surgery and endovascular treatment in a large series, there was no significant difference in the complication rates and 30- and 90-day mortality rates [29]. In recent years, endovascular treatment of PAA using multi-layer flow-modulating stents has been shown to be a safe and feasible method [30,31].

Nonetheless, there are some limitations to this study. Small sample size and nonrandomized, retrospective design are the main limitations. In addition, patients requiring emergency intervention due to acute limb ischemia were excluded from the study. Therefore, no results were obtained regarding the performance of the graft used in this patient group. There are also some prerequisites, such as diameter compliance, tortuosity, and angulation suitability that should not be required in the surgical treatment of popliteal artery aneurysms but should be provided in the treatment of endovascular stent grafts. 

## 4. Conclusion

In conclusion, endovascular treatment of PAA using appropriate stent grafts is safe in selected cases who are at high risk for surgery due to comorbidities or those who refuse surgery. It is also advantageous owing to its shorter operation duration and length of hospital stay, more rapid functional recovery, minimal blood loss and wound site infection, and potential avoidance of general anesthesia. The distal vascular bed and distal and proximal attachment regions must be carefully examined before endovascular treatment. It is reasonable to avoid endovascular treatment due to an increased risk for thrombosis in patients with a low number of patent distal arteries or impaired distal flow. In addition, PAAs may present with concomitant AAA and patients should be evaluated for possible AAAs. Limb salvage is possible with a second endovascular intervention even in patients with graft thrombosis.

## Informed consent

The necessary approval for our study has been granted by the Ordu Provincial Health Directorate on the date of July 6th, 2020 with the protocol no 0012038785.

## References

[ref1] (2015). Two decades of endovascular repair of popliteal artery aneurysm. A meta-analysis. European Journal of Vascular and Endovascular Surgery.

[ref2] (2007). Surgical and endovascular treatment of atherosclerotic popliteal artery aneurysms. The Journal of Cardiovascular Surgery (Torino).

[ref3] (2005). Arterial aneurysms. In: Cronenwett JL (editor).

[ref4] (1997). Atherosclerotic popliteal aneurysm. The British Journal of Surgery.

[ref5] (2010). M Clinical outcome of acute leg ischemia due to thrombosed popliteal artery aneurysm: systematic review of 895 cases. European Journal of Vascular and Endovascular Surgery.

[ref6] (2014). Endovascular treatment of aneurysms of the popliteal artery by a covered endoprosthesis. Clinical Medicine Insights. Cardiology.

[ref7] (1994). Transfemoral endoluminal stented graft repair of a popliteal artery aneurysm. Journal of Vascular Surgery.

[ref8] (2007). A contemporary review of popliteal artery aneurysms. Cardiology in Review.

[ref9] (2007). Mid-term outcomes of endovascular popliteal artery aneurysm repair. Journal of Vascular Surgery.

[ref10] (1995). The thrombosed popliteal artery aneurysm and distal arterial occlusion: successful therapy by interdisciplinary management. The Thoracic and Cardiovascular Surgeon.

[ref11] (2011). Part one: for the motion asymptomatic popliteal artery aneurysms (less than 3 cm) should be treated conservatively. European Journal of Vascular and Endovascular Surgery.

[ref12] (2013). Update on aneurysm disease: current insights and controversies: peripheral aneurysms: when to intervene-is rupture really a danger? Progress in Cardiovascular Diseases.

[ref13] (1983). Limb – threatening potential of arteriosclerotic popliteal artery aneurysms. Surgery.

[ref14] (2005). Open repair versus endovascular treatment for asymptomatic popliteal artery aneurysm: results of a prospective randomized study. Journal of Vascular Surgery.

[ref15] (2015). Treatment of popliteal aneurysm by open and endovascular surgery a contemporary study of 592 procedures in Sweden. European Journal of Vascular and Endovascular Surgery.

[ref16] (2015). Comparison of popliteal artery aneurysm therapies. Journal of Vascular Surgery.

[ref17] (2006). Endovascular treatment of popliteal artery aneurysms. Vascular.

[ref18] (2009). Endovascular exclusion of popliteal artery aneurysms with stent-grafts: a prospective single-center experience. Journal of Endovascular Therapy.

[ref19] (2014). Long-term outcomes and sac volume shrinkage after endovascular popliteal artery aneurysm repair. European Journal of Vascular and Endovascular Surgery.

[ref20] (2013). Heparin-bonded covered stents versus bare-metal stents for complex femoropopliteal artery lesions: the randomized VIASTAR trial (Viabahn endoprosthesis with PROPATEN bioactive surface (VIA) versus bare nitinol stent in the treatment of long lesions in superficial femoral artery occlusive disease). Journal of the American College of Cardiology.

[ref21] (2006). Endovascular repair of popliteal artery aneurysms: techniques,current evidence and recent experience. Australian and New Zealand Journal of Surgery.

[ref22] (2012). S et al. Journal of Vascular Surgery.

[ref23] (2015). Comparison of popliteal artery aneurysm therapies. Journal of Vascular Surgery.

[ref24] (2005). Endovascular treatment of popliteal artery aneurysms: results of a prospective cohort study. Journal of Vascular Surgery.

[ref25] (2005). Popliteal aneurysms: distortion and size related to symptoms. Journal of Vascular and Endovascular Surgery.

[ref26] (2007). Dall’Antonia A et al. The Journal of Cardiovascular Surgery (Torino).

[ref27] (2012). A Retrospective multicenter study of endovascular treatment of popliteal artery aneurysm. Journal of Vascular Surgery.

[ref28] (2013). Evolving treatment of popliteal artery aneurysms. Journal of Vascular Surgery.

[ref29] (2013). Endovascular versus open repair of popliteal artery aneurysms: outcomes in the US Medicare population. European Journal of Vascular and Endovascular Surgery.

[ref30] (2018). Early and mid-term results in the endovascular treatment of popliteal aneurysms with the multilayer flow modulator. Vascular.

[ref31] (2020). Endovascular treatment of popliteal artery aneurysms: A report of three cases. Turkish Journal of Vascular Surgery.

